# On the move: sloths and their epibionts as model mobile ecosystems

**DOI:** 10.1111/brv.12773

**Published:** 2021-07-26

**Authors:** Maya Kaup, Sam Trull, Erik F. Y. Hom

**Affiliations:** ^1^ Department of Biology and Center for Biodiversity and Conservation Research University of Mississippi University MS 38677‐1848 U.S.A.; ^2^ The Sloth Institute Tulemar Gardens, Provincia de Puntarenas Manuel Antonio 60601 Costa Rica

**Keywords:** symbiosis, mutualism, algae, fungi, arthropods, hair, fur, microbiome, epibiont, movement ecology

## Abstract

Sloths are unusual mobile ecosystems, containing a high diversity of epibionts living and growing in their fur as they climb slowly through the canopies of tropical forests. These epibionts include poorly studied algae, arthropods, fungi, and bacteria, making sloths likely reservoirs of unexplored biodiversity. This review aims to identify gaps and eliminate misconceptions in our knowledge of sloths and their epibionts, and to identify key questions to stimulate future research into the functions and roles of sloths within a broader ecological and evolutionary context. This review also seeks to position the sloth fur ecosystem as a model for addressing fundamental questions in metacommunity and movement ecology. The conceptual and evidence‐based foundation of this review aims to serve as a guide for future hypothesis‐driven research into sloths, their microbiota, sloth health and conservation, and the coevolution of symbioses in general.

## I. INTRODUCTION

**Fig 1:**
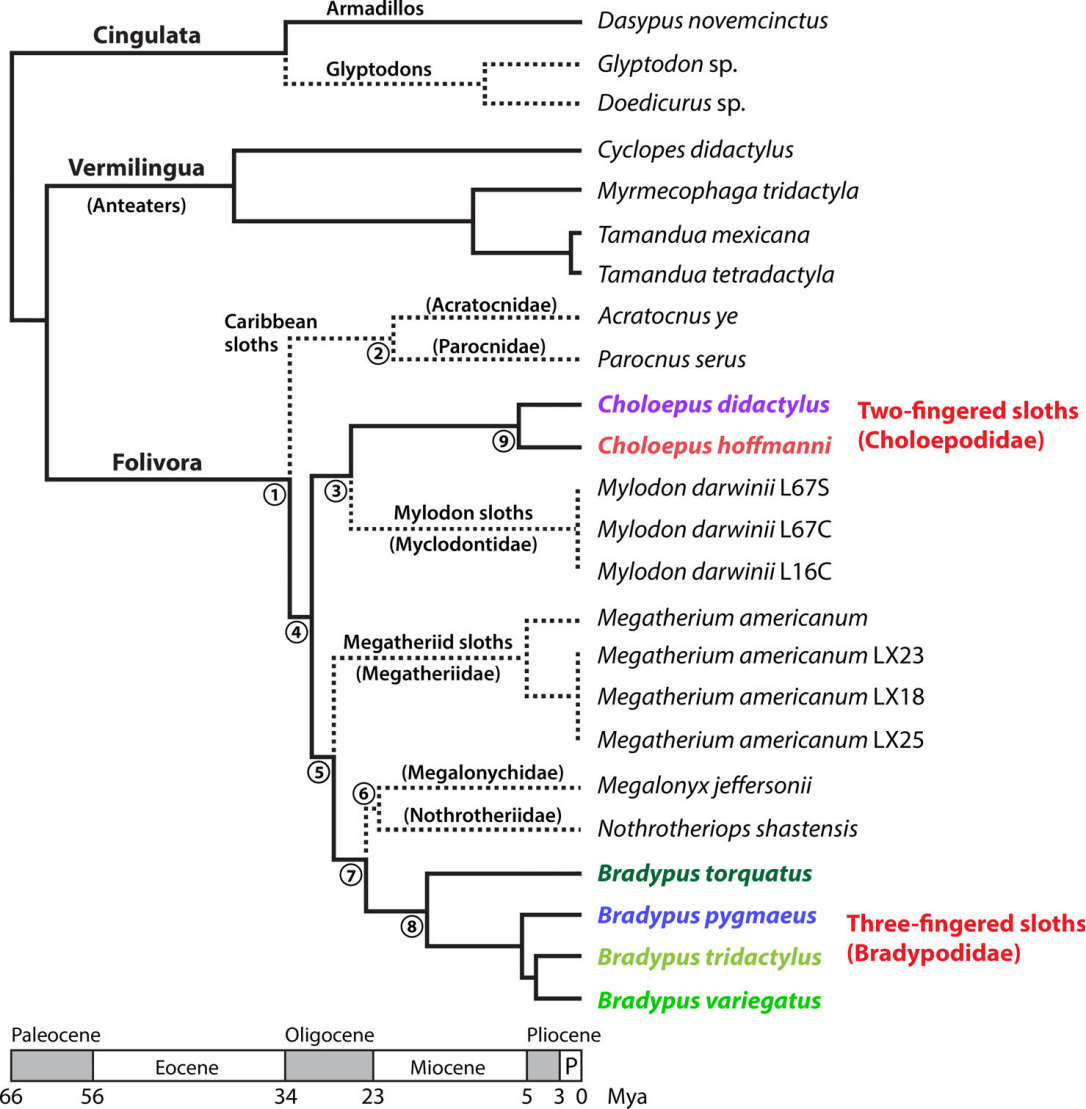
Phylogeny of sloths and their xenarthran relatives, anteaters and armadillos, with approximate timescales for branches. Dashed lines indicate extinct lineages or species. Main geological periods are shown (P = Pleistocene). Timescales are in millions of years (Mya). Synthesized from Delsuc *et al*. (2019) and Presslee *et al*. (2019), with indicated branch point timings being the averages of those reported by these two studies: (1) 33 Mya, (2) 21 Mya, (3) 26 Mya, (4) 31 Mya, (5) 27 Mya, (6) 22 Mya, (7) 24 Mya, (8) 17 Mya, and (9) 6 Mya. Recent molecular phylogenetic data suggests that *Bradypus torquatus* may be better assigned to a different genus (*Scaeopus*) and that *Bradypus variegatus* may represent two distinct species (trans‐Andean and cis‐Andean), although further studies are needed to clarify formal species distinctions (Ruiz‐García *et al*., 2020).

Sloths spend much of their lives hanging from trees in Central and South America and are unique in that they have some of the slowest metabolisms of all mammals (Pauli *et al*., 2016). There are six extant species of sloths in two genera: two‐fingered (*Choloepus* spp., family Choloepodidae) and three‐fingered (*Bradypus* spp., family Bradypodidae) (Slater *et al*., 2016). Historically, the names ‘two‐toed’ and ‘three‐toed’ have been used, although this is a misnomer; we use ‘two‐fingered’ and ‘three‐fingered’ herein because all sloths have three toes but differ in the number of ‘fingers’ they have on their upper limbs. Despite both genera being slow‐moving arboreal folivores, two‐ and three‐fingered sloths are very different as revealed by molecular, morphological, and behavioural data (Table 1). Mitogenome and ancient collagen DNA phylogenetic analyses have revealed that these two sloth genera diverged ~31 million years ago (Fig. 1; Delsuc *et al*., 2019; Presslee *et al*., 2019). Both sloth genera host an array of largely unexplored symbioses (i.e. persistent, physical associations; Bronstein, 2015) involving taxonomically diverse microorganisms and arthropods in a multi‐trophic assemblage within their fur or pelage (Aiello, 1985; Gilmore, da Costa & Duarte, 2001; Suutari *et al*., 2010; Higginbotham *et al*., 2014). The structure of sloth hair is also unusual, being characterized by cracks or grooves that are hypothesized to facilitate algal growth (Aiello, 1985; Suutari *et al*., 2010), giving them a distinct green coloration in the wild.

**Table 1:** Comparison of two‐ and three‐fingered sloth characteristics. Synthesized from Aiello (1985), Anderson & Handley (2001), Britton (1941), Chiarello (2008), Falconi *et al*. (2015), Feldhamer *et al*. (2015), Goodwin & Ayres (2014), Higginbotham *et al*. (2014), Mendoza *et al*. (2015), Montgomery & Sunquist (1978), Nie *et al*. (2015), Nyakatura (2012), Pauli & Peery (2012), Pauli *et al*. (2014, 2016), Peery & Pauli (2012), Ramirez *et al*. (2011), Sunquist & Montgomery (1973), Taube *et al*. (2001), Urbani & Bosque (2007), Vaughan *et al*. (2007), and Wetzel (1985). It should be noted that *Choloepus hoffmanni* and *Bradypus variegatus* home range sizes were based largely on observations in mixed‐cacao plantation agroecosystems and thus may not truly represent native home ranges for these species (Montgomery & Sunquist, 1978; Vaughan *et al*., 2007; Ramirez *et al*., 2011). Predicted home range values (*H*_*pred*_) are based on Jetz *et al*.'s (2004) scaling relation for mammalian herbivores: (1.02 ± 0.9 ha/kg) × *M +* (2.05 ± 0.5 ha), where *M* is sloth body weight (kg); upper and lower values were calculated using extreme upper and lower bounds of input values

Two‐fingered sloths	Three‐fingered sloths
***Gross anatomy/morphology***	
Modified, hook‐like arms and feet	Modified, hook‐like arms and feet
Rounded thorax with a small diameter	Rounded thorax with a small diameter
Relatively long arms with a relatively short scapula	Relatively long arms with a relatively short scapula
High mobility of all joints proximal to the midcarpal and transverse tarsal joints	High mobility of all joints proximal to the midcarpal and transverse tarsal joints
Highly mobile sterno‐clavicular articulation	Highly mobile sterno‐clavicular articulation
Powerful flexion in the proximal limb joints *via* advantageous lever arms	Powerful flexion in the proximal limb joints via advantageous lever arms
Two forelimb fingers	Three forelimb fingers
5–8 neck vertebrae	8–9 neck vertebrae
Body mass: up to 8.5 kg	Body mass: up to 6 kg
*C. hoffmanni: mean (sd) = 5.7 ± 0.7 kg*	*B. variegatus: mean = 4.3 ± 0.9 kg*
*C. didactylus: mean (sd) = 6 ± 1 kg*	*B. torquatus: range = 4.1–6.0 kg*
	*B. tridactylus: mean = 4.0 ± 0.3 kg*
	*B. pygmaeus: range = 2.5–3.5 kg*
Similar limb length	Forelimbs longer than hindlimbs
No tail	Small tail
Caniniform premolars	Only cylindrical teeth
***Physiology and diet***	
Diet is mostly leaves, but also fruits, eggs, and insects	Diet is almost exclusively leaves
*C. hoffmanni*: third slowest metabolism of all mammals (energy expenditure = 234 kJ/d/kg)	*B. variegatus*: slowest metabolism of all mammals (energy expenditure = 162 kJ/d/kg)
10‐month gestation	5–6‐month gestation
***Behaviour and range***	
Suspensory, arboreal locomotion	Suspensory, arboreal locomotion
No basking behaviour	Basking behaviour
Vigorous self‐defence	Minimal self‐defence
Nocturnal	Cathemeral (sporadic activity over 24 h), although *B. torquatus* observed to be nocturnal in warmer temperatures
Promiscuous	Polygynous
Movements (*C. hoffmanni*):	Movements (*B. variegatus*):
>50% travel ≥38 m per day	~90% travel <38 m per day
<10% on same tree in successive days	<40% on same tree in successive days
Total activity per day: 7.6 ± 1.5 h	Total activity per day: 10.1 ± 2.2 h
Continuous bouts of activity:	Continuous bouts of activity:
<1 h, 45%	<1 h, 66%
1–2 h, 29%	1–2 h, 19.5%
*2–6 h, 23%	*2–6 h, 13%
6–10 h, 3%	6–10 h, 1.5%
*accounts for majority (52%) of total time active	*accounts for majority (43%) of total time active
Home range:	Home range:
*C. hoffmanni:*	*B. variegatus:*
median = 4.4–7.5 ha	median = 5.2 ha
male mean (sd) = 9 ± 53 ha	male mean (sd) = 22 ± 57 ha
female mean (sd) = 6 ± 9 ha	female mean (sd) = 2 ± 25 ha
*H_*pred*_ = 6.7–9.2 ha*	*H_*pred*_ = 5.2–7.8 ha*
*C. didactylus: unknown; H_*pred*_ = 6.7–9.9 ha*	*B. torquatus: median = 6.6 ha; range = 0.4–22 ha*
	*H_*pred*_ = 5.9–8.7 ha*
	*B. tridactylus: unknown; H_*pred*_ = 5.5–6.9 ha*
	*B. pygmaeus: unknown; H_*pred*_ = 4.3–6.0 ha*
***Fur‐related***	
Visible algal growth on hair, four known genera	Visible algal growth on hair, six known genera
Fungal genera unclear	16 fungal genera identified
Longitudinal hair grooves	Transverse hair cracks

With a complex and largely self‐contained community of interacting epibionts defined by a boundary layer of fur, the sloth holobiont (host + associated biota) likely functions as an ecological unit in space (Jax, 2006). As sloths move slowly through the greater forest ecosystem, they can be considered ‘mobile ecosystems’ or dynamic, moving islands of biodiversity (*cf*. Heaney, Balete & Rickart, 2013; Borregaard *et al*., 2017). As such, they could represent unique systems to investigate questions in host–epibiont/host–microbiome ecology and coevolution within an unusual spatiotemporal/movement regime not typical of sessile organisms or fast‐moving animals.

We aim to highlight how studying sloths and their epibionts may be useful in addressing fundamental questions in microbial and metacommunity ecology, movement ecology, microbiome science, and the evolution of symbioses. We summarize what is known about the basic biology of sloths as it relates to their epibionts, and review evidence (or lack thereof) in support of several speculative conclusions that have accrued in the literature that have unfortunately led to misconceptions now canonized in the popular media (Meier, 2013; Greenwood, 2014; Lewis, 2014; Woollaston, 2014). We aim to challenge speculations that lack clear empirical support, articulate gaps in our understanding of the sloth as a mobile ecosystem (focused particularly on sloth fur as an ecosystem), and make suggestions for future directions in sloth research.

## II. THE SLOTH AS A MODEL MOBILE ECOSYSTEM

All animals possess an assemblage of other species that live on or within them, the majority of which are microbial. When found within (as with gut microbiomes) these species often have a profound influence on host biology (McFall‐Ngai, 2015; Barko *et al*., 2017). As for other mammals, the gut microbiome of sloths is believed to play an important role in sloth health and be influenced by diet (Delsuc *et al*., 2014; Dill‐McFarland *et al*., 2016). However, it is the rich diversity of epibiotic symbionts on sloth fur that is most distinctive about the sloth holobiont and the focus of this review. Unlike the gut microbiome, which is shielded from the environment except through host‐driven dietary intake, the sloth fur ecosystem is open to the larger forest ecosystem through which the sloth moves. In addition to microbes, a variety of arthropods are an integral part of the fur multi‐trophic community (discussed in Section III). Similar to the pitcher plant (Boynton, 2012; Bittleston *et al*., 2018; Miller, Bradshaw & Holzapfel, 2018*b*), which contains an elaborate food web of predators, prey, and detritivores that reside within a leafy ‘cup’ and is an entire ecosystem unto itself, sloth fur is a relatively self‐contained system of taxonomic diversity and trophic levels.

How a sloth's fur is colonized by this biodiversity is unknown but the process may be strongly influenced by the ecology of the skin/hair, endogenous host factors, and exogenous environmental factors as in humans (Grice & Segre, 2011). As with other host‐microbiome systems, the ‘extended phenotype’ (Dawkins, 1982) of the sloth could impose an ecological filter that shapes what and how epibionts assemble (Stagaman *et al*., 2017; Henry *et al*., 2019; Gilbert *et al*., 2020). The sloth fur ecosystem may have a ‘layered’ organization, similar to the canopy structure of a species‐rich grassland (Lane, Coffin & Lauenroth, 2000) or the stratified communities of microbial mats (Stolz, 2000) in which organisms are organized based on gradients in temperature or light penetration. The closer to hair follicles and skin, the warmer, dimmer, and more stable local conditions may be compared to those at the ends of hair tips that are more exposed to the elements. The sloth fur microbiome may be the foundation for recruiting and assembling taxa from higher trophic levels (discussed in Section IV.1) and may be fundamental to the well‐being of the sloth (discussed in Section IV.2). Like trees that are colonized by microbes in their phyllosphere (leaves) (Vacher *et al*., 2016) and by lichens in their dermosphere (bark) (Lambais, Lucheta & Crowley, 2014), slow‐moving sloths may be reservoirs of similar types of microbes that colonize substrates with low levels of movement‐based disturbance. Moreover, as sloths move from tree to ground and tree to tree in the forest canopy (Montgomery & Sunquist, 1975; Vaughan *et al*., 2007; see online Supporting Information, [Link], [Link]), they may acquire epibionts from interacting with hundreds of species of trees, each of which host unique phyllosphere, dermosphere, and rhizosphere communities (Lambais *et al*., 2014; Leff *et al*., 2015). As discussed further in Section IV.3, sloths may be vectors of dispersal unlike any other animal and provide a unique opportunity to understand ecological and evolutionary processes at a spatiotemporal scale that links microbes, arthropods, animal host movements, and three‐dimensional forest structure (*cf*. Prosser *et al*., 2007; Antwis *et al*., 2017; Shade *et al*., 2018).

### (1) Unique sloth traits

Two‐ and three‐fingered sloths have many similar traits (Table 1), some that are hypothesized to have evolved convergently. Both groups of sloths have evolved suspensory posture, long, sharp claws for gripping branches and for territorial fights, and a modified skeletal structure to suit their slow, arboreal lifestyle (Miller, 1935; Montgomery & Sunquist, 1978; Mendel, 1981, 1985; Nyakatura & Fischer, 2011; Nyakatura, 2012; Pauli *et al*., 2016; Olson *et al*., 2018). Suspensory posture, and the many anatomical adaptations that have arisen for efficient suspensory locomotion in trees (see Supplementary Videos S3 and S4), are the most clearly convergent traits, given that no known fossil sloths were considered suspensory (Nyakatura, 2012). Evidence for a convergently evolved slow metabolism and diet is lacking, however. While it is not clear if ground sloths had cracked/grooved hair, this is a distinctive trait of all extant sloth species and has not been found on the closest relatives of sloths, armadillos and anteaters (Aiello, 1985; discussed further in Section III.1*a*), nor for any other mammal. The only other known mammals with epibiotic algal growth are polar bears in zoos (Lewin & Robinson, 1979) and manatees (Bledsoe *et al*., 2006), although they do not appear to have hair with crevices.

Sloths have diets consisting largely of leaves from trees, with three‐fingered sloths being almost exclusively folivorous. All sloths have low basal metabolic rates as is common for all arboreal folivores dependent on nutritionally poor food sources (McNab, 1978, 1986). Hoffmann's two‐fingered sloth, *Choloepus hoffmanni*, has the third slowest metabolism (234 kJ/day/kg) of all mammals, with a daily energy expenditure slightly greater than another folivorous mammal, the giant panda, *Ailuropoda melanoleuca* (185 kJ/day/kg) (Nie *et al*., 2015; Pauli *et al*., 2016). The brown‐throated three‐fingered sloth, *Bradypus variegatus*, has the slowest metabolism (162 kJ/day/kg; Pauli *et al*., 2016) and slowest rate of digestion of any mammal (Foley, Engelhardt & Charles‐Dominique, 1995).

### (2) Geographical range, movement, and behaviour

**Fig 2:**
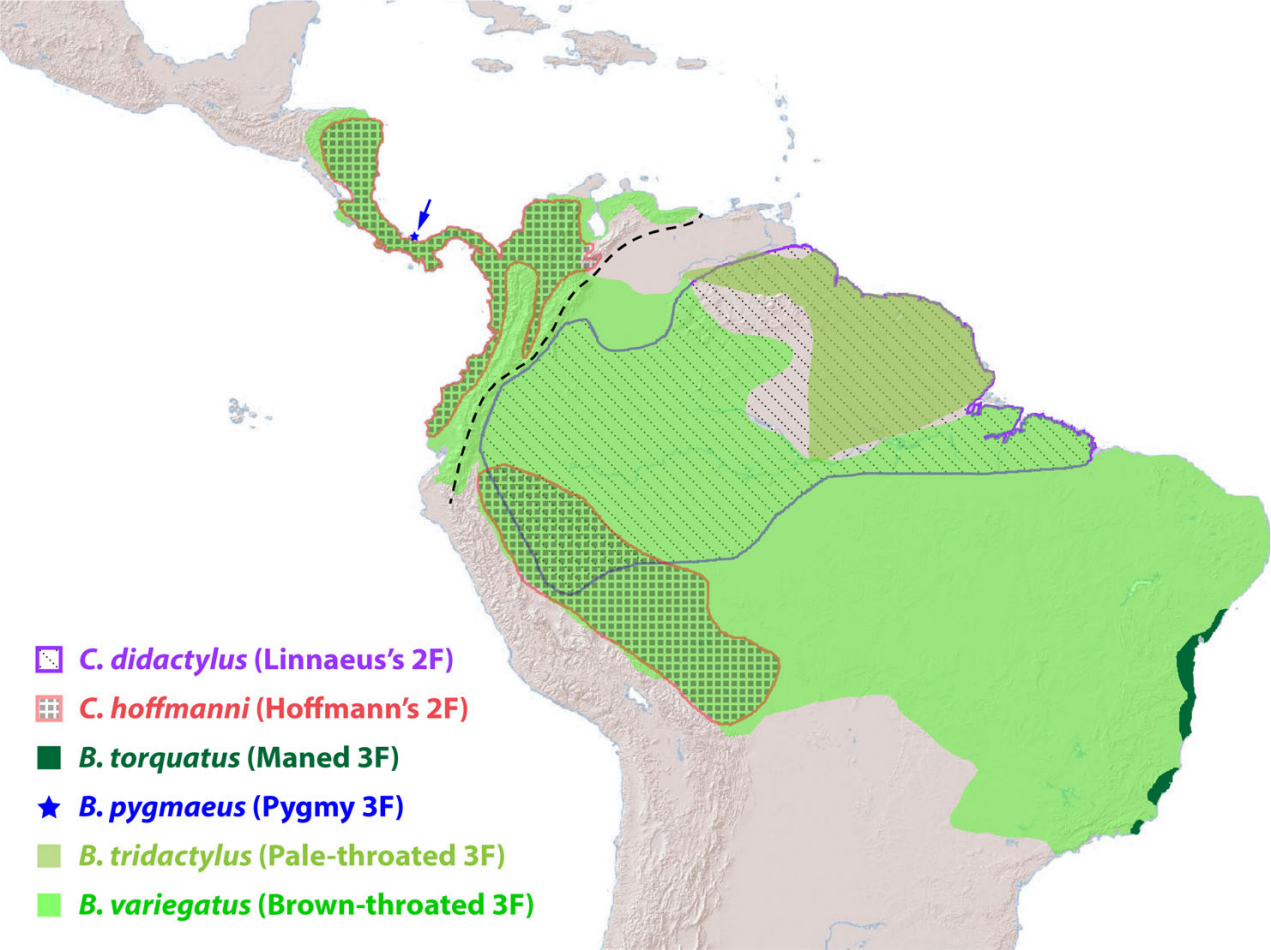
Distributional range of extant two‐fingered (2F) and three‐fingered (3F) sloth species across Central and South America. Synthesized from data of Chiarello & Plese (2014), Plese & Chiarello (2014), Chiarello & Moraes‐Barros (2014*a*,*b*), Voirin *et al*. (2014) and Moraes‐Barros, Chiarello & Plese (2014) available at https://www.iucnredlist.org/. The population of trans‐Andean *B. variegatus* sloths located north and west of the Cordillera Oriental mountain range in Colombia (dashed line) is believed to represent a distinct species (*Bradypus ephippiger*) although this awaits formal confirmation (Ruiz‐García *et al*., 2020).

Geographically, sloths are found throughout the neotropical forests of Central and South America, occupying a native range from Guatemala south through Peru and Brazil (Fig. 2). Although it is commonly thought that sloths are quite sedentary, they have been observed to move regularly throughout the forest canopy at rates of up to 0.5 km/h in short bursts (Sunquist & Montgomery, 1973). In one study based on radio‐telemetry observations, 54% of *C. hoffmanni* sloths were observed to move ≥38 m between daily locations (Sunquist & Montgomery, 1973) and <10% of observed sloths were found on the same tree on successive days. These two‐fingered sloths are nocturnal with the majority of their activity occurring in bouts lasting 2–6 h, with total activity averaging 7.6 h per day (Table 1). In a cacao agroecosystem, *C. hoffmanni* was found in 101 different tree species and eating from 34 species (Vaughan *et al*., 2007). By contrast, *B. variegatus* sloths tended to travel shorter distances, with 89% observed to travel *<*38 m per day (Sunquist & Montgomery, 1973). Unlike *C. hoffmanni*, these three‐fingered sloths are active throughout the day and night and for a greater proportion of the day (~10 h) but are four times more likely to remain on the same focal tree on successive days (Sunquist & Montgomery, 1973). This is consistent with observations of *B. variegatus* being found in and eating from fewer tree species than *C. hoffmanni* (71 and 15, respectively), and their preference to return more frequently to feed on specific trees, especially *Cecropia* spp. (Vaughan *et al*., 2007; Neam & Lacher, 2015; Garcés‐Restrepo, Peery & Pauli, 2019*b*). *B. variegatus* is more likely to have bouts of activity of shorter duration than *C. hoffmanni*, with the majority being <1 h (Table 1). Like *C. hoffmanni*, however, bouts of activity lasting 2–6 h each account for the majority of the total time active during the day.

Home range sizes for sloths varies depending on species and sex, estimates of which are based largely on observations of *C. hoffmanni* and *B. variegatus* in mixed‐use landscapes centered around cacao plantations (see Table 1). Median home ranges for *C. hoffmanni* and *B. variegatus* sloths are estimated to be 4–7.5 and ~5 ha, respectively, which are generally within estimates predicted by a body size–space use scaling law for mammalian herbivores (Jetz *et al*., 2004; Table 1). However, individual home range observations are extremely variable, with a long‐tail distribution (and mode <2 ha) (Sunquist & Montgomery, 1973; Vaughan *et al*., 2007; Peery & Pauli, 2012); these data were typically acquired over a 1‐year period and it is possible that range estimates could increase (and variability decrease) with longer observation times (*cf*. Vaughan *et al*., 2007). Nonetheless, males appear to have larger home ranges than females in general.

Based on what we know about *C. hoffmanni* and *B. variegatus*, sloths move across the forest not just laterally, but up and down the canopy column. Sloths favour floristically diverse and structurally complex forest structures (vertically and horizontally), with dense canopy cover and variable tree heights (Montgomery & Sunquist, 1978; Mendoza *et al*., 2015; Neam & Lacher, 2015, 2018). As heterotherms, they prefer to rest and feed high up in the forest canopy (Neam & Lacher, 2015), moving in and out of canopy shade/sunlight and up and down trees to regulate their body temperatures as needed depending on ambient temperatures throughout the day (Montgomery & Sunquist, 1978; Pauli *et al*., 2016).

Sloths also exhibit the unusual behaviour of descending all the way down to the ground to defecate about once a week (Montgomery & Sunquist, 1975; Waage & Montgomery, 1976; Montgomery & Sunquist, 1978; Voirin *et al*., 2013). Many ideas have been proposed to explain this unusual defecation behaviour. It has been proposed that defecating on the ground (as opposed to letting dung drop from the canopy of trees) is a strategy that sloths use to remain undetected, since being quiet and hidden seems to be their predominant life strategy and defecating from the canopies of trees presumably may cause a disturbance that attracts predators (S. Trull, unpublished data). However, there is no evidence that descending to the base of the tree is risky to the sloth, especially since the majority of their predators, harpy eagles (*Harpia harpyja*), spectacled owls (*Pulsatrix perspicillata*), ocelots (*Leopardus pardalis*), and tayra (*Eira barbara*), can also detect and attack them from the tree canopy, often by knocking them to the ground where they proceed to eat them (Beebe, 1926; Izor, 1985; Bezerra *et al*., 2009; Voirin *et al*., 2009). Other theories include proposed benefits from fertilizing their most frequently used trees, communicating with other sloths through social latrines, trying to hide their scent from predators, sustaining a three‐way mutualism with moths and algae (discussed further in Section III.2*c*), or deriving nutritional benefits from consuming soil while on the ground (Beebe, 1926; Krieg, 1939; Goffart, 1971; Voirin *et al*., 2013). However, observational data suggest that three‐fingered sloths do not frequently eat soil (S. Trull, unpublished data), and no data exist in support of the other theories.

## III. COMPONENTS OF THE MOBILE ECOSYSTEM

### (1) Algae

**Fig 3:**
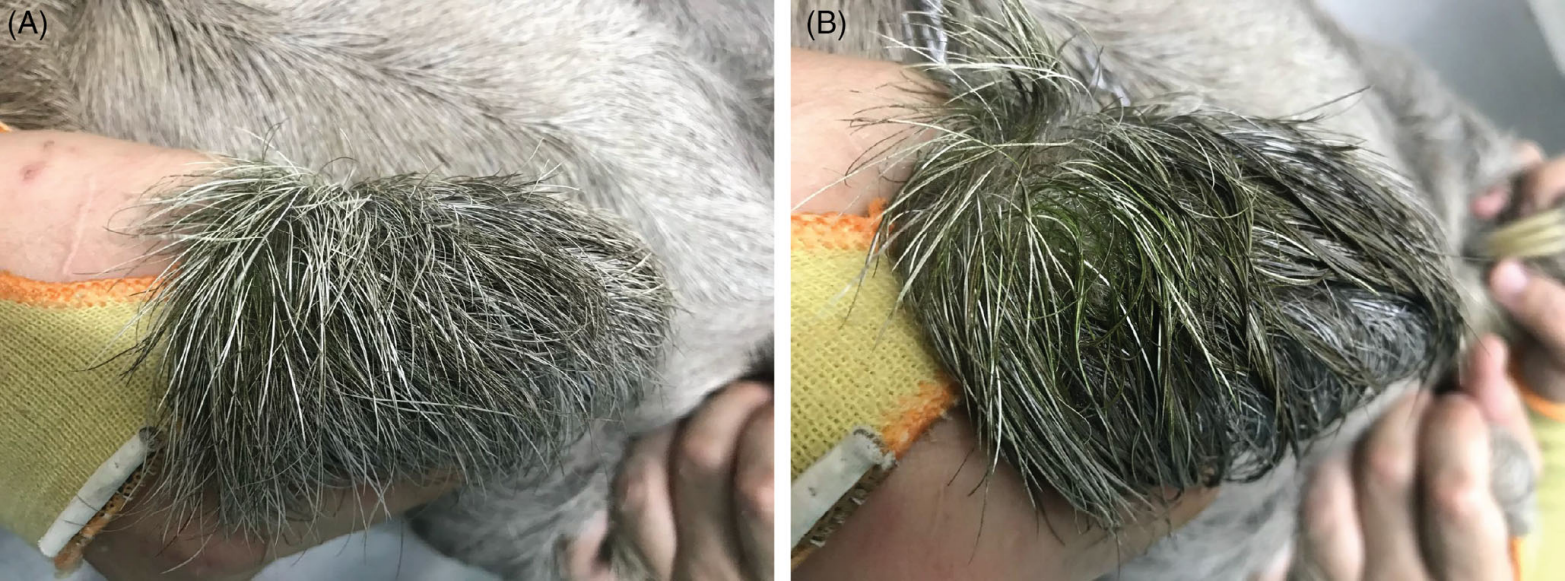
Dry and wet sloth hair. Hair on the back of the hand of (A) a dry *Bradypus variegatus* (brown‐throated three‐fingered) sloth, and (B) the same hand 10 s after wetting reveals a rapid greening and the presence of otherwise visually cryptic green algae/cyanobacteria.

The green hue of sloths arises from green algae that grow on sloth hair (Aiello, 1985; Suutari *et al*., 2010). Cyanobacteria may also contribute to this greenish hue, although only one species, *Oscillatoria pilicola*, has been identified to the species level to date (Table 2; Wujek & Lincoln, 1988). DNA sequences for red algae have also been found on sloths (Table 2; Suutari *et al*., 2010). For this review, we use the term ‘algae’ to refer broadly to eukaryotic algae and cyanobacteria unless specifically distinguished. It is not clear if algae are resident on all sloths in the wild. One study found that 73% of the 74 sampled sloths had visible algae on their fur identified *via* eye or microscope [*Bradypus variegatus* (*N* = 18), *Bradypus tridactylus* (*N* = 12), *Bradypus pygmaeus* (*N* = 12), *Bradypus torquatus* (*N* = 8), *Choloepus hoffmanni* (*N* = 22), *Choloepus didactylus* (*N* = 2)] (Suutari *et al*., 2010). However, neither sloth age, season of sampling, nor location were accounted for, and included in this analysis were captive sloths from zoos, which may lack native epibionts (likely due to being bred in captivity, bathed, or being kept in an enclosed habitat away from potential microbial symbionts in their native habitat). Given the limited and uneven sampling of this study, this value of 73% should not be interpreted as a definitive statistic. It is also generally overlooked that ‘brown’ sloths may actually host epibiotic algae even though not visibly green to the naked eye (Goffart, 1971): such algae may simply be in a dormant or non‐green state when moisture is limited. In fact, wetting of ‘brown’ sloth hair results in a rapid greening within seconds to minutes (Fig. 3), akin to what is observed with the wetting of desiccated biological soil crusts (Abed *et al*., 2014; Pietrasiak, 2014).

**Table 2:** Known descriptions of algae found in sloth fur. Descriptions derived from Friedl (1995)^a^, Printz (1964)^b^, Schubert (2003)^c^, Suutari *et al*. (2010)^d^, Wujek & Timpano (1986)^e^, or AlgaeBase.org (Guiry & Guiry, 2019)

Genus	Phylum	Class	Description
*Trichophilus*	Chlorophyta	Ulvophyceae	Small (3–13 μm) thick‐walled cells with numerous, small, discoid chloroplasts that lack pyrenoids^b,d^
*Trentepohlia*	Chlorophyta	Ulvophyceae	Filamentous, orange in colour
*Pseudendoclonium*	Chlorophyta	Ulvophyceae	Filamentous, marine, cells with single parietal chloroplast and a pyrenoid
*Trichosarcina*	Chlorophyta	Ulvophyceae	Filamentous, cells with single parietal chloroplast and pyrenoid
*Ulothrix*	Chlorophyta	Ulvophyceae	Unbranched filaments with cells always closely adherent, uninucleated cylindrical cells
*Printzina*	Chlorophyta	Ulvophyceae	Filamentous, uninucleated cells, chloroplasts parietal and band‐shaped
*Collinsiella*	Chlorophyta	Ulvophyceae	Gelatinous, uninucleated cells, cup‐shaped chloroplasts
*Asterochloris*	Chlorophyta	Trebouxiophyceae	Found in association with fungus in lichen, single asteroid chloroplast in a crenulate, echinate, or lobed form
*Chlorella*	Chlorophyta	Trebouxiophyceae	Cells spherical, subspherical or ellipsoid, single or forming colonies, chloroplast single, parietal, pyrenoid present
*Nannochloris*	Chlorophyta	Trebouxiophyceae	Subspherical to subcylindrical, 0.8–4.5 μm in diameter unicells. May occur in pairs enclosed in mucilage, or in large numbers in a mucilage mass^c^
*Trebouxia*	Chlorophyta	Trebouxiophyceae	Found in association with fungus in lichen, pyrenoid present
*Stichococcus*	Chlorophyta	Trebouxiophyceae	Unbranched filaments, cell walls thin, without gelatinous sheath, cells cylindrical and elongate, sometimes slightly oval
*Myrmecia*	Chlorophyta	Trebouxiophyceae	Coccoid cells, found in association with lichenous fungi; not to be confused with the genus of ants by the same name^a^
*Dictyococcus*	Chlorophyta	Chlorophyceae	Zoospores with a single parietal plastid nearly closed and lacking a pyrenoid, spherical cells^e^
*Chlorococcum*	Chlorophyta	Chlorophyceae	Uninucleated cells, ellipsoidal to spherical and varying in size, cell walls smooth, parietal chloroplast and with one or more pyrenoids
*Planophila*	Chlorophyta	Chlorophyceae	Uninucleated cells, spherical, solitary or tightly grouped in small (usually 2–8 cells) colonies, thin cell walls
*Oscillatoria*	Cyanobacteria	Cyanophyceae	Filamentous, trichomes blue‐green to brownish‐green, highly motile
*Nostoc*	Cyanobacteria	Cyanophyceae	Filamentous‐thallose, gelatinous, cells cylindrical, barrel‐shaped to almost spherical
*Fischerella*	Cyanobacteria	Cyanophyceae	Filamentous‐thallose, thallus usually felt‐like, usually barreliform cells
*Rufusia*	Rhodophyta	Stylonematophyceae	Branched‐filamentous, several parietal, discoidal to band‐shaped plastids with no pyrenoid, reddish to violet in colour

#### (a) Sloth hair structure and algal growth

**Fig 4:**
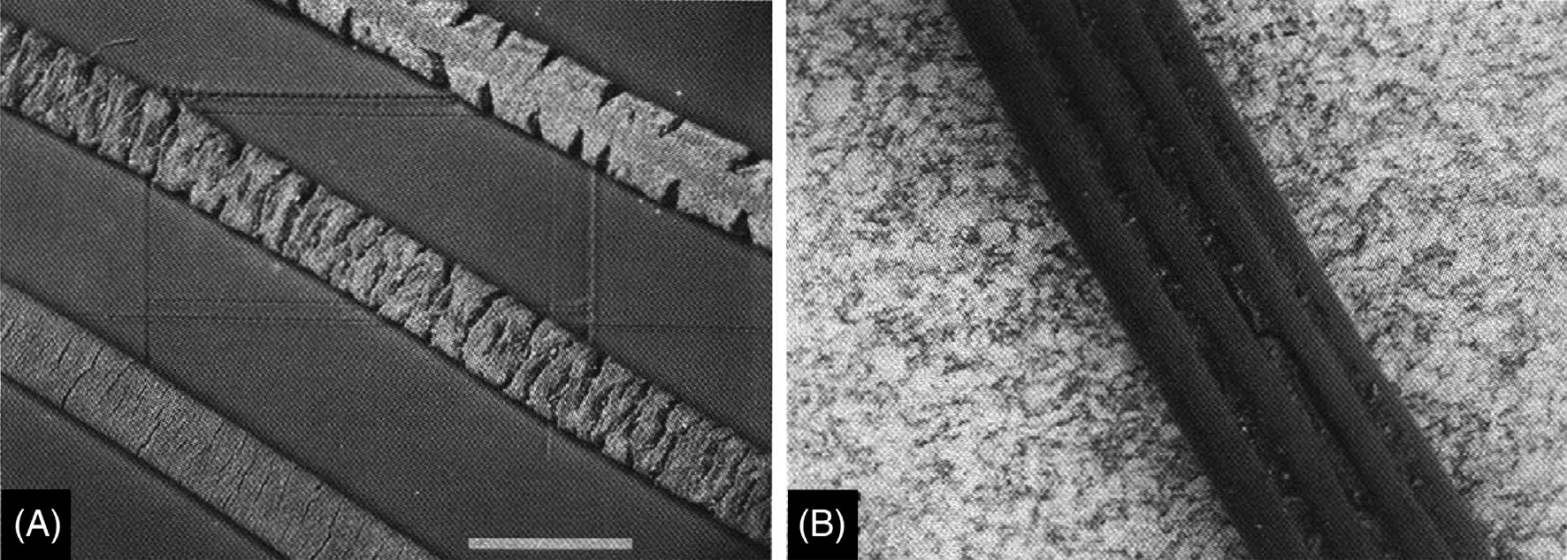
Scanning electron micrographs of sloth hairs. (A) *Bradypus variegatus* (brown‐throated three‐fingered sloth) hair at three different stages of development (scale bar = 0.6 mm). The bottom hair is from a young sloth in which transverse cracks are only beginning to develop. The middle hair is from an adult sloth displaying larger cracks. The top hair is from an old sloth and shows deep transverse cracks. (B) *Choloepus hoffmanni* (Hoffmann's two‐fingered sloth) hair showing longitudinal ribs or grooves, at 6× higher magnification than in A. Photographs reproduced from Aiello (1985) with permission (Smithsonian Institution Press).

The morphology of sloth hair has the potential to influence the extent and composition of symbiotic growth. Three‐fingered sloth hair has transverse cracks that increase in quantity and depth as sloths age (Fig. 4; Aiello, 1985; Wujek & Cocuzza, 1986). The hairs swell considerably when wet, and it has been hypothesized that moisture that is retained within cracks sustains algal growth on the surface of the hairs (Aiello, 1985). It has also been hypothesized that this hair‐based absorption of water may help buffer changes in sloth body temperature (Pauli *et al*., 2016). It does not appear that the algae grow directly within the cracks, which would potentially limit access to photosynthetic radiation, but rather grow on the smooth outer surface of the hair (Aiello, 1985). It remains unknown whether algae directly colonize hair strands with pre‐existing cracks and/or if they contribute to hair crack development. By contrast, two‐fingered sloth hair has vertical grooves and does not appear to absorb as much water; algae appear only to be found within the grooves instead of coating the entire hair (Fig. 4B; Aiello, 1985; Wujek & Cocuzza, 1986). Differences in hair architecture may be responsible for the observed differences in fur amplicon sequencing surveys between the two genera of sloths (Aiello, 1985; Suutari *et al*., 2010). Although increased absorptive properties due to unusual hair structure are not limited to sloths (Kingdon *et al*., 2012), the unique cracked/grooved hair structure of sloths seems to facilitate symbiotic algal growth unlike any other mammal (Aiello, 1985). It is unknown whether algal and fungal species typically found on sloth hair are able to grow on texturally smooth hair. Whether such hair cracks/grooves co‐evolved with the associated microbes or is a convergently evolved trait among two‐ and three‐fingered sloths remains an open question.

#### (b) Identification of sloth algae

Morphological identification of sloth algae has yielded confusing results; in most cases, the sloth species from which algae have been derived was not recorded (Table 2). In a conference abstract by Thompson (1972), many sloth‐fur‐associated algae and cyanobacteria were listed, identified *via* morphology. However, algal and cyanobacterial species can be very hard to distinguish morphologically; DNA‐ and polyphasic‐based methods are typically required to make clear taxonomic assignments (Leliaert *et al*., 2014; Wilmotte *et al*., 2017). Unfortunately, no follow‐up confirmations of Thompson's (1972) identifications exist in the literature and Thompson did not specify from which sloth species these algae were obtained. Thompson identified two species of *Oscillatoria* and one of *Nostoc*, but it is not clear if either of these *Oscillatoria* are the same as the *Oscillatoria pilicola* identified and described by Wujek & Lincoln (1988) on both the fur of *B. variegatus* and *C. hoffmanni*. The genus *Fischerella*, three coccoid green algae (including *Dictyococcus bradypodis* and *Chlorococcum choloepodis*), three species of *Trentepohlia*, two of *Stichococcus*, and one of *Nannochloris* were identified (Table 2; Thompson, 1972; Wujek & Timpano, 1986). *Rufusia*, a red alga named by Wujek & Timpano (1986), was also identified on both *B. variegatus* and *C. hoffmanni*.

**Fig 5:**
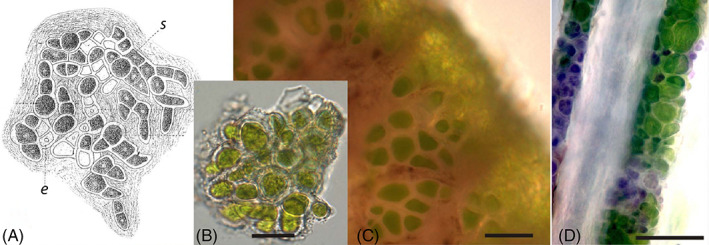
Morphology of green algal clusters, presumably of *Trichophilus welckeri*, found in sloth hair. (A) *Trichophilus welckeri* ‘fronds’ as described by Weber‐van Bosse (1887, fig. 15). s, sporangia; e, empty sporangial cells. (B, C) *Trichophilus*‐like alga from a hair of the pygmy three‐fingered sloth, *Bradypus pygmaeus*. (D) Hair with *Trichophilus*‐like alga from a Hoffmann's two‐fingered sloth, *Choloepus hoffmanni*. Modified from a figure in Suutari *et al*. (2010). Scale bars, 20 μm.

*Trichophilus welckeri*, the best‐known sloth green alga, was first identified on sloths in 1887 (Weber‐van Bosse, 1887). *Trichophilus* is in the class Ulvophyceae and is characterized by small (3–13 μm) thick‐walled cells with numerous, small, discoid chloroplasts that lack pyrenoids (Fig. 5; Table 2; Printz, 1964; Suutari *et al*., 2010). While solely morphology‐based taxonomic identifications must be taken cautiously, many of the aforementioned findings seem to be unknown to most modern readers of the sloth literature. Nonetheless, it appears that the diversity of green algae and cyanobacteria may be far greater than is suggested by more recent studies that focus on *T. welckeri* and its role in the sloth hair ecosystem (Pauli *et al*., 2014). Other species of algae should be taken into consideration, however, to understand how the community of photobionts is functioning and impacting its accompanying fungal and bacterial epibionts, arthropods, and the sloth itself.

Amplicon metagenomic studies of sloth fur to date reveal a variable array of algae across and within different sloth species. Using amplicon‐based techniques, *T. welckeri* was found on the fur of *B. variegatus*, the pale‐throated sloth, *Bradypus tridactylus*, and the pygmy three‐fingered sloth, *Bradypus pygmaeus* (Suutari *et al*., 2010). No other green algal species were identified on these sloths from this 18S amplicon sequencing study (Suutari *et al*., 2010; Table 3) despite the prior observations by Thompson (1972), Wujek & Timpano (1986) and Wujek & Lincoln (1988). This discrepancy may be due in part to the use of captive sloths from zoos that potentially lack native epibionts (as mentioned earlier) and/or the intrinsic limitations of the use of 18S ribosomal DNA (rDNA) as a barcode for resolving algal taxa (Hall *et al*., 2010). *T. welckeri* has not yet been found environmentally (Suutari *et al*., 2010), although this may be a consequence of insufficient environmental sampling across the sloths’ geographical range and within the canopies of trees. The maned three‐fingered sloth, *Bradypus torquatus*, hosts a variety of algae belonging to genera known to be terrestrial, e.g. *Trentepohlia* and *Myrmecia* (Table 3; Suutari *et al*., 2010). *C. hoffmanni*, and *B. tridactylus* host *Trichophilus* spp. as well as terrestrial green algae from their surroundings (Table 3; Suutari *et al*., 2010).

**Table 3:** Sloth species and associated algal epibionts identified to date. Those with an asterisk following the genus have thus far only been found on sloths and not yet on other environmental substrates. Data are from Suutari *et al*. (2010; clarified in some cases through personal correspondence with M. Suutari and J. Blomster). Cyanobacteria are indicated by superscript ^C^. Eleven genera not listed in the table, *Chlorococcum*, *Collinsiella*, *Dictyococcus*, *Fischerella* ^C^, *Nannochloris*, *Nostoc* ^C^, *Planophila*, *Pseudendoclonium*, *Stichococcus*, *Trichosarcina*, and *Ulothrix*, were found on sloths, but are of an unidentified origin (Thompson, 1972; Wujek & Timpano, 1986). Note that *Myrmecia* below is a genus of green algae associated with lichens, not the genus of ants

Sloth common name	Scientific name	Algal genera
Brown‐throated three‐fingered sloth	*B. variegatus*	Trichophilus*, *Oscillatoria* ^C^, *Rufusia*
Pygmy three‐fingered sloth	*B. pygmaeus*	Trichophilus*
Pale‐throated three‐fingered sloth	*B. tridactylus*	Trichophilus*
Maned three‐fingered sloth	*B. torquatus*	*Trentepohlia*, *Myrmecia*, *Asterochloris*, *Chlorella*, *Printzina*, *Trebouxia*
Hoffmann's two‐fingered sloth	*C. hoffmanni*	Trichophilus*, *Oscillatoria* ^C^, *Rufusia*, *Trentepohlia*
Linnaeus's two‐fingered sloth	*C. didactylus*	No data

The 18S sequences for *Trichophilus* spp. found in association with *B. variegatus*, *B. pygmaeus* and *B. tridactylus* were found to cluster separately from *Trichophilus* sequences obtained from *C. hoffmanni* (Suutari *et al*., 2010). *Trichophilus* spp. from *Bradypus* and *Choloepus* differ in cell size, and *B. variegatus* and *T. welckeri* phylogenies are consistent with codivergence, which has led some to propose that *B. variegatus* and *T. welckeri* have coevolved (Suutari *et al*., 2010; Fountain *et al*., 2017). However, matching phylogenies is an insufficient demonstration of reciprocal coevolution (Janzen, 1980; Anderson, 2015). The differences in hair structure discussed earlier may impact differential colonization of sloth hair and the poorly characterized biogeography of environmental sources of sloth algae might explain the underlying phylogenetic concordance.

#### (c) Algal benefits

**Fig 6:**
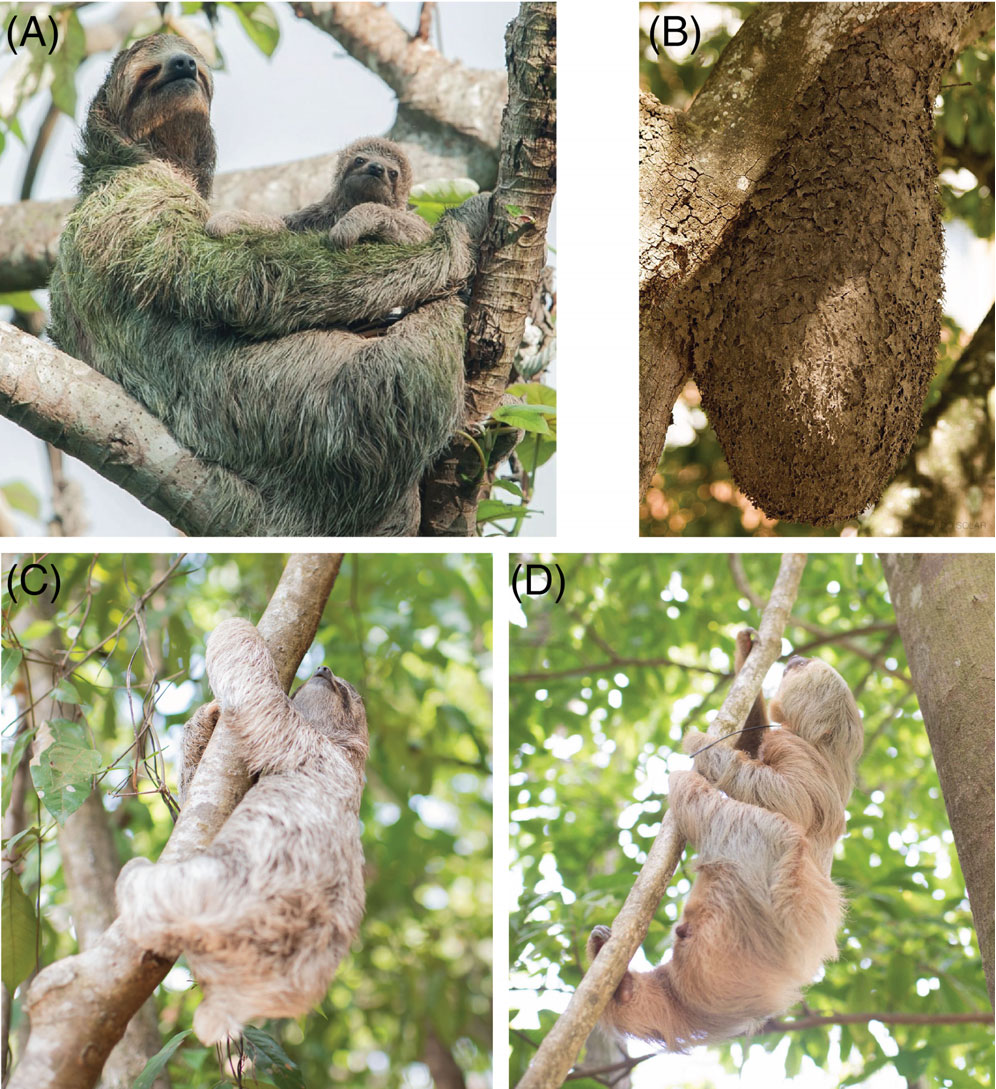
Colour and shape similarities of sloths. (A) A female *Bradypus variegatus* (brown‐throated three‐fingered sloth) with green fur coloration, taken during the wet season; (B) an Azteca ant carton nest that looks similar to a hanging sloth; (C) a dry *B. variegatus* sloth and (D) a dry *Choloepus hoffmanni* (Hoffmann's two‐fingered) sloth with similar coloration as the branches, vines, and bark of the trees they inhabit. Photograph of Azteca ant nest by Solar (2014) used with permission under Creative Commons License CC BY‐NC‐SA 2.0.

Several hypotheses have been proposed for how algae might benefit sloths, however, they all lack concrete empirical support, and in fact, it is not clear if algae provide any benefit to sloths. It is possible that it is simply a commensal relationship, and sloths may have so much algae in their fur because they do not have the means to clean themselves. Despite this, it is widely believed that fur algae provide a camouflage benefit to the sloth (Aiello, 1985; Suutari *et al*., 2010; Pauli *et al*., 2014), but no studies have been pursued to test this hypothesis. As discussed above, sloth fur coloration can change: they are primarily green during the rainy season when their hair is regularly wet (Fig. 6A), and in the dry season, many sloths lose their greenish hue and appear brown or grey (Britton, 1941; Gilmore *et al*., 2001). Direct observations of brown/grey sloths in their native canopy suggest that they are very well camouflaged with this colour scheme, blending in with the branches, trunks, and dead leaves of trees (Fig. 6C, D), as well as resembling ant and termite nests (Fig. 6B; Goffart, 1971).

Fur algae have also been proposed to serve as a nutritional food source for sloths based on remnants of green algal cells being found in their stomach contents (Pauli *et al*., 2014). However, there is little evidence to support the idea that sloths ‘farm’ algae on their fur that they then consume, or that they consume sufficient quantities of fur algae to be nutritionally beneficial (discussed further in Section III.2*c*). Other proposed hypotheses in the literature for how algae could benefit sloths include: (*i*) algae being a source of thermal insulation (Britton & Atkinson, 1938; Goffart, 1971; Montgomery & Sunquist, 1978; Aiello, 1985); (*ii*) algae providing some yet unidentified chemical benefit to overall sloth health (Aiello, 1985); (*iii*) algae producing exopolymeric substances to facilitate beneficial bacterial growth (Suutari *et al*., 2010); and (*iv*) algae acting as a sunscreen (Suutari *et al*., 2010). *T. welckeri* has been found to produce an UV‐absorbing mycosporine‐like amino acid believed to protect algae from UV radiation (Karsten *et al*., 2005); whether this protection extends to sloths with *T. welckeri* on their fur (beyond the intrinsic UV‐protection of fur itself) is unknown. To our knowledge, none of these hypotheses are supported by any evidence‐based rationale.

### (2) Arthropods

#### (a) Biting arthropods

Sloths are also host to a wide range of arthropods living in their fur including parasitic, bloodsucking and biting arthropods such as mosquitoes and sandflies, triatomine bugs, lice, mites, and ticks (Gilmore *et al*., 2001). Six species of ticks have been found on two‐ and three‐fingered sloths, all from the genus *Ambylomma*, but only two species, *Ambylomma geayi* and *Ambylomma varium*, appear specialized for living on sloths as these ticks are rarely found on other hosts (Waage & Best, 1985). Tick infestation can be extremely high. At the Instituto Nacional de Pesquisas da Amazonia in Manaus (Brazil), 99% of three‐fingered and 86.7% of two‐fingered sloths carried *Ambylomma* spp. (Waage & Best, 1985). Nothing is known about how *A. geayi* or *A. varium* find a host sloth and no correlation has been found between the numbers of ticks at any life stage on a sloth or seasonal differences in rainfall (Gilmore *et al*., 2001). The blood‐sucking mites, *Liponissus inheringi*, *Lobalges trouessarti*, and *Edentalges bradypus* have been identified on three‐fingered sloths (Waage & Best, 1985) and the mite *Edentalges choloepi* has been found on Linnaeus's two‐fingered sloth, *Choloepus didactylus* (Fain, 1964). Whether ectoparasite loads impact the health of a sloth is an open question.

#### (b) Commensals and beetles

**Fig 7:**
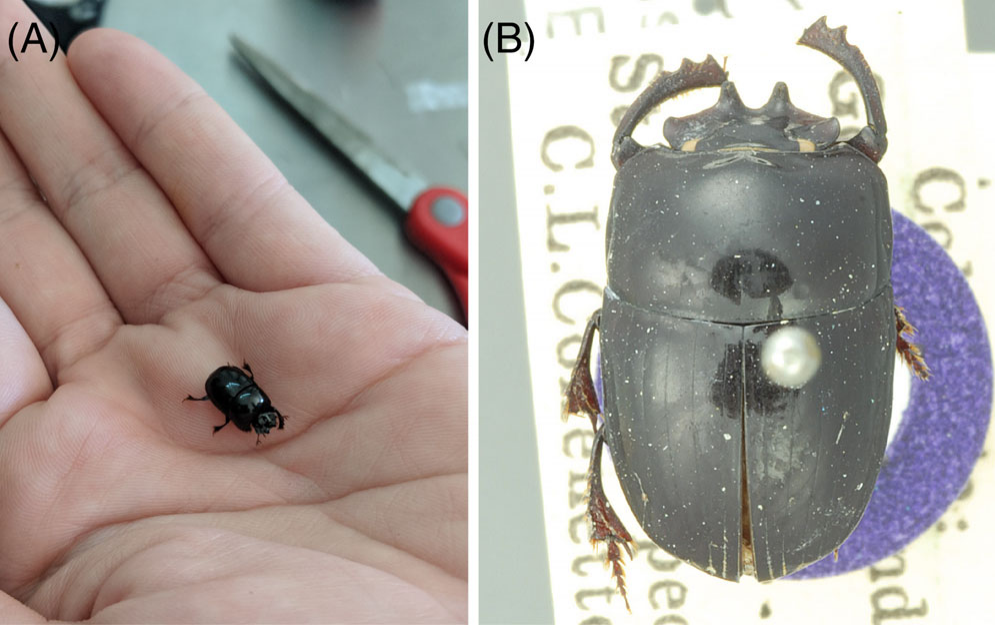
The sloth‐associated scarab beetle “*Uroxys gorgon* Arrow, 1933.” (A) Collected live from the fur of a *Bradypus variegatus* (brown‐throated three‐fingered sloth), and (B) a mounted specimen (Larsen, 2015), used with permission under Creative Commons License CC BY‐NC 3.0.

Many commensal arthropods are found in association with these slow‐moving mammals. It is quite possible that the algae on sloth fur serves as a food source for these commensal arthropods considering that mites and other insects display algophagy (Mckenna *et al*., 2015; Seniczak *et al*., 2016). Cockroaches have been found in sloth fur (Britton, 1941), although this may be quite rare (S. Trull, unpublished data). Adults of several scarab beetle species are frequently found in the fur of three‐fingered sloths (of which the beetle in Fig. 7 is an example), but have not been reported to be associated with *Choloepus* (Ratcliffe, 1980; Gilmore *et al*., 2001). The scarab beetles occur near the elbow or on the flanks behind the knees, buried deep inside the fur. The beetles found living on sloths are considered commensal because they are phoretic coprophages: the beetle larvae (and possibly adults) feed on sloth dung and they do not appear to harm the sloths (Ratcliffe, 1980; Gilmore *et al*., 2001). About a thousand such beetles (*Trichillum adisi*) have been found in the fur of a single brown‐throated three‐fingered sloth (*B. variegatus*) collected on Curari Island in the Central Amazon region (Waage & Best, 1985). Beetles of the genus *Uroxys* have been recorded from sloths in Bolivia, Brazil, Colombia and Panama (Waage & Best, 1985). Despite the ubiquity of beetle–sloth interactions, little is known about the dispersal and density fluctuations of these beetles on sloths, although in Panama, there seem to be higher numbers of beetles during the rainy season (Wolda & Estribi, 1985). It has been suggested that the beetles have dispersal flights at the beginning and end of the rainy season and that part of the population might enter reproductive diapause and disperse from the sloths to sites with some moisture; they presumably resume reproduction at the end of the dry season and return to the sloths (Wolda & Estribi, 1985). Just as there are no data to substantiate an effect of parasite load on sloths, no analysis has been performed to understand the effect of these suspected commensal arthropods or of total arthropod load on sloth health. Likewise, little is known of the potential role these beetles might play in the ecosystem. It is possible that beetles contribute to parasite suppression, secondary seed dispersal, and to nutrient cycling within the sloth fur ecosystem and the larger forest ecosystem (Nichols *et al*., 2008). It is also possible that some sloth‐associated arthropods play a protective and mutualistic role by preying on ectoparasites in sloth fur (*cf*. Ostlund‐Nilsson, Becker & Nilsson, 2005; Goedknegt, Welsh & Thieltges, 2012).

#### (c) Sloth moths

**Fig 8:**
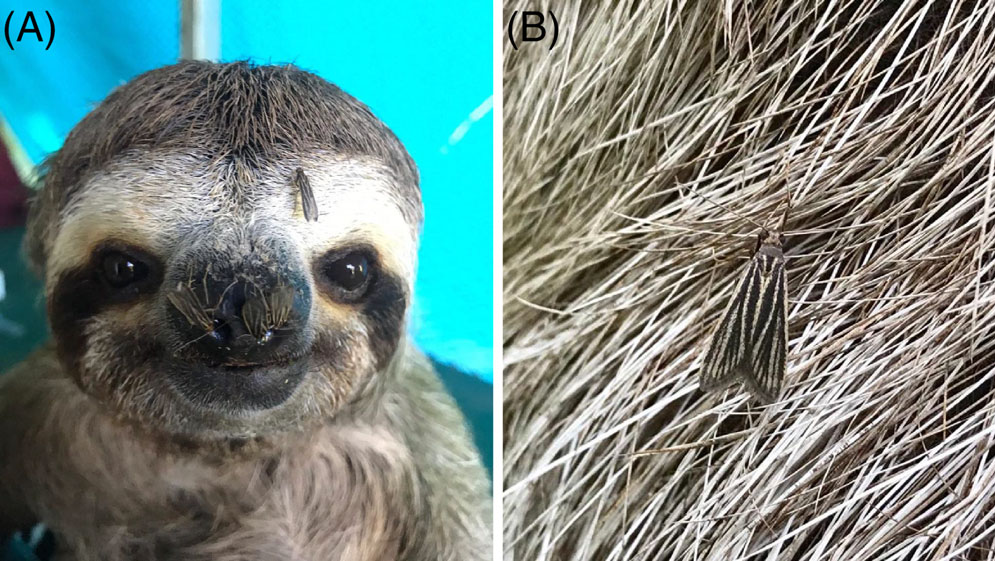
The sloth moth, *Cryptoses choloepi*, on a *Bradypus variegatus* (brown‐throated three‐fingered sloth). (A) Moths often swarm the sloth's face, especially orifices such as the nose and eyes, and (B) appear well camouflaged on the sloth's grey‐brown fur.

Sloth moths in the genus *Cryptoses* have received notable attention as a sloth epibiont. There is appreciable geographic sympatry amongst sloth‐associated moth species and several different species may coexist in the fur of a single sloth (Waage & Best, 1985). Various sloth moth species appear to be found on all species of sloths (Bradley, 1982; Waage & Best, 1985; Pauli *et al*., 2014). *Cryptoses choloepi* seems to be the most common moth found on *B. variegatus* and has been studied almost exclusively in relation to this sloth species (Fig. 8; Supplementary Videos S2 and S3). Female *C. choloepi* moths that live in *B. variegatus* fur have been observed to oviposit in the dung of the sloth as the sloth descends to the forest floor to defecate. Moth larvae in early stages spin silken threads between two or three pellets of dung, forming net‐like structures from which they feed (Waage & Montgomery, 1976). Upon maturation after 3–4 weeks, newly emerged moths presumably fly from the dung pile into the forest canopy to find a new sloth host (Waage & Montgomery, 1976). In addition to nutritional benefits that sloth moth larvae might receive from feeding on sloth dung, adult moths may consume sloth/algal secretions or microbes on sloth hair (Fig. 8); the sloth moth gut microbiome has yet to be explored but may provide evidence for this. Adult moths are believed to receive a transportation benefit as well as a protection benefit from living in sloth fur (Waage & Montgomery, 1976; Wolda, 1985). The amount of protection moths receive in association with sloths is questionable, however, since brown jays (*Psilorhinus morio*) have been observed to consume insects off sloth fur (Neam, 2015).

Based on studies to date, it would appear that sloth moths have a commensal relationship with their sloth hosts. However, a three‐way mutualism has been proposed involving *B. variegatus*, their moths, and fur algae, specifically *T. welckeri*. According to this hypothesis, moths are portals for nutrients, increasing nitrogen levels in sloth fur through defecation, which is believed to promote algal growth (Pauli *et al*., 2014). *T. welckeri*‐like algae have been found (microscopically) in sloth stomach contents, which has led to the hypothesis that sloths consume these algae to augment their limited diet (see Section III.1*c*). With this set of observations, the proposal is that sloths are involved in an evolutionary trade‐off in which they risk their lives, descending to the ground to defecate, in order to preserve this sloth–moth–algae tripartite mutualism (Pauli *et al*., 2014). There are at least five potential problems with this hypothesis. First, the method of identifying the alga in this study as *T. welckeri* by morphology alone is not sufficiently rigorous, especially given that this taxon is often morphologically cryptic and under‐studied in general (Dudgeon *et al*., 2017). Second, while the main groups of bacteria that inhabit the gut microbiome of *B. variegatus* have been identified (Dill‐McFarland *et al*., 2016), no metagenomic study to date has been performed to characterize or confirm the eukaryotic/algal diversity in this species’ gastrointestinal tract. Sloths have been observed to lick and eat material off of branches and tree trunks, which may include lichens (Tirler, 1966; S. Trull, unpublished data), thus the algae observed in the stomachs of sloths may have derived from environmental substrates rather than from their fur. Third, thousands of hours of sloth behavioural research recorded during the day and night do not support the idea that sloths lick themselves (like cats) or eat epibiotic algae from their fur (Tirler, 1966; S. Trull, unpublished data). Fourth, only two *B. variegatus* individuals out of 12 sampled in one location in Costa Rica were identified as having *Trichophilus* spp. in their stomachs (Pauli *et al*., 2014). And lastly, if sloth tree‐descent and ground‐defecation is driven by a need to benefit moths *via* dung oviposition, one would expect there to be reciprocal fitness benefits provided to the sloth by the moths in order for this behaviour to have evolved or be maintained (Voirin *et al*., 2013); however, the implied and indirect benefits that sloths might obtain from moth‐influenced fur algal growth lack empirical support and may be quantitatively modest.

### (3) Fungi

Fungi are known to be associated with sloth hair, but the roles they play in the community ecology of the sloth pelage and in the health of the sloth remain unexplored. A diverse group of Ascomycota and one Basidiomycete (*Sporobolomyces subbrunneus*) have been identified growing on sloth fur through sequencing and culture‐based methods (Suutari *et al*., 2010; Higginbotham *et al*., 2014). Only two species of fungi that have been found on sloths have also been found on the bark of trees in sloth habitats (*Devriesia staurophora* and *Mycosphaerella pini*; Suutari *et al*., 2010), although these results are from very limited sampling. These sloth‐associated fungi have been found in soil and plants (Arnold & Lutzoni, 2007; Wang *et al*., 2011), so it is possible that sloths are exposed to these fungi when they defecate on the ground or as they eat and interact with leaves and bark (Higginbotham *et al*., 2014). Nearly 35% of fungal isolates obtained from *B. variegatus* fur are identical to endophyte strains obtained from plants in the same region (Higginbotham *et al*., 2014). Given the taxonomic similarity between endolichenic and endophytic plant fungi in the same environments (U'ren *et al*., 2012), it is plausible that some sloth hair fungi may associate directly with green algae (Higginbotham *et al*., 2014). Previous studies support that fungi, and these taxa in particular, have intrinsic affinities for associating and forming mutualisms with algae, as seen in lichens [typically slow growing and commonly found on undisturbed trees (Hawksworth, 1988; Arnold *et al*., 2009)], and other similar symbioses (Hawksworth, 2000; Gareth Jones, Pang & Stanley, 2012; Hom & Murray, 2014; Du *et al*., 2019).

**Fig 9:**
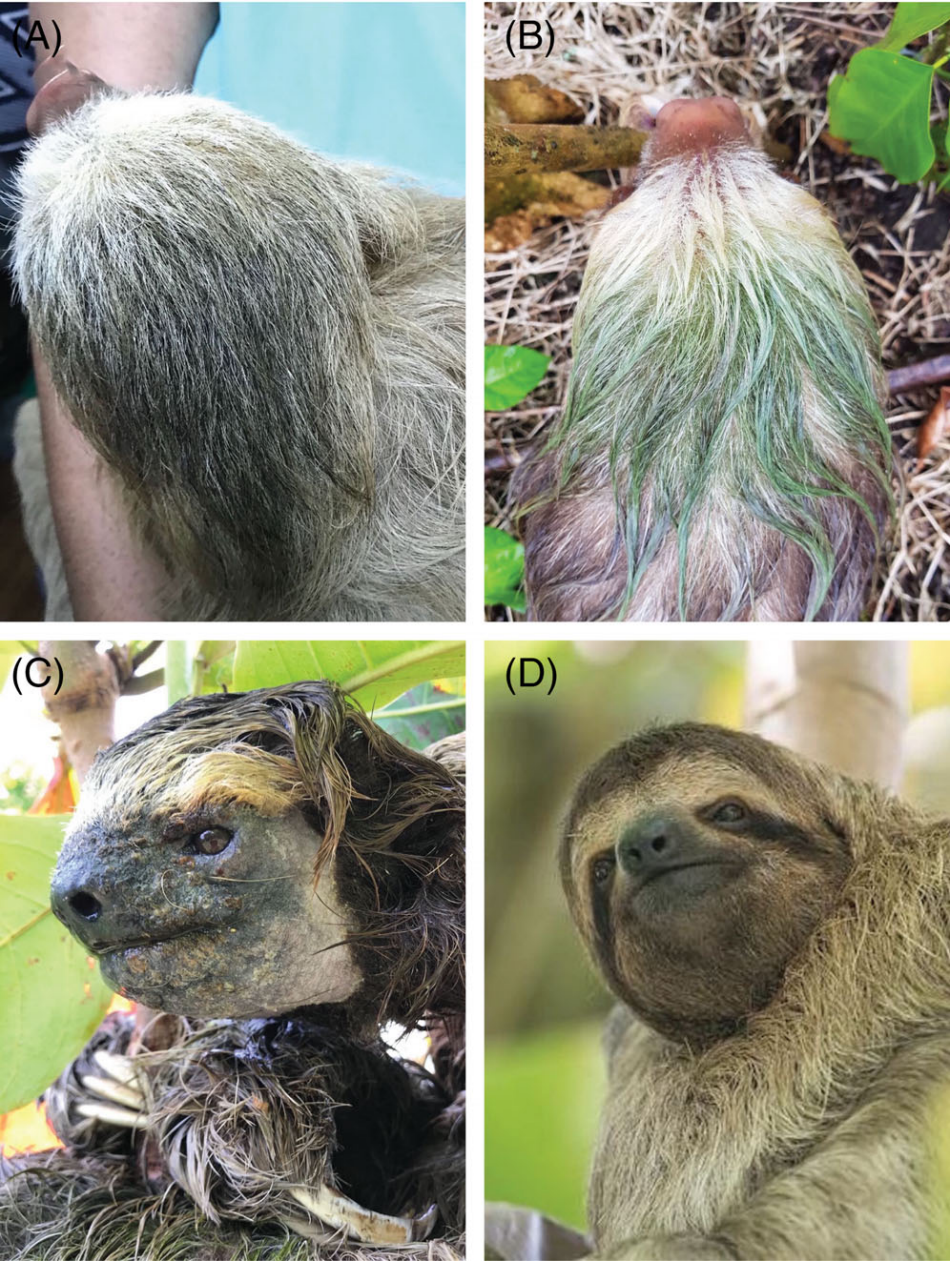
Sloths and fungi. (A, B) The back of the heads of two *Choloepus hoffmanni* (Hoffmann's two‐fingered sloth) with visible growth on the fur of (A) black fungi and (B) algae. (C, D) Facial photographs of (C) a *Bradypus variegatus* (brown‐throated three‐fingered sloth) with a severe fungal infection that causes scabs of hair to fall off, and (D) a healthy *B. variegatus* sloth for comparison.

However, whether sloth‐associated fungi are commensals, parasitic, or mutualistic is not clear. Hair‐associated fungi from *B. variegatus* have been shown to display a broad range of inhibitory activities against parasites that cause malaria (*Plasmodium falciparum*) and Chagas disease (*Trypanosoma cruzi*), human breast cancer cells, and bacteria, particularly Gram‐negative bacteria (Higginbotham *et al*., 2014). The inhibitory activities of these fungi may thus provide benefits to the sloth as well. Some sloths have clear black fungal growth on their hair (Fig. 9A), which could potentially harm the sloth or outcompete other microbes in the sloth hair ecosystem. Others develop severe fungal infections on their skin that can be detrimental because the infections produce scabs, which then fall off, leaving bare skin that is susceptible to ectoparasites like ticks and mosquitos (Fig. 9C; Xavier *et al*., 2008); anecdotally, fungal infections generally correlate with sick sloths (S. Trull, unpublished data). It is unclear whether these infections derive from fungi that are already part of the sloth fur microbiome and/or whether they are a primary cause for sloths getting sick.

### (4) Other symbionts

In addition to the algae, arthropods, and fungi that live and thrive within the pelage of sloths, other putative fur‐associated organisms have been identified through 18S amplicon sequencing; these include euglenozoans, amoebozoans, cercozoans, apicomplexans, dinoflagellates, and ciliates (Table 4; Suutari *et al*., 2010). To date, nothing is known about the role of these organisms within the sloth hair ecosystem. Apart from the sloth fur cyanobacteria mentioned above (Table 2), fur‐associated prokaryotes have not been well documented or sufficiently taxonomically resolved. Surprisingly, a 16S survey of the bacterial diversity on sloth fur has not yet been performed; it will be important to survey the prokaryotes present in the sloth fur ecosystem and to understand the inter‐kingdom interactions they may have with the sloth and other fur epibionts. Bacterial epibionts may influence the function of sloth‐associated fungi and algae, as they do for fungal endophytes associated with plants (Partida‐Martinez & Hertweck, 2005; Hoffman & Arnold, 2010) and lichens (Grube & Berg, 2009; Bates *et al*., 2011).

**Table 4:** Other epibionts found in sloth fur. Species names were assigned based on the closest known matches in GenBank. Percentage similarity is to the closest match in GenBank. Data from Suutari *et al*. (2010). Given the low similarity for most matches and little taxonomic follow‐up, these species designations may not be correct

Phylum	Taxon	Percentage similarity
Euglenozoa	*Petalomonas cantuscygni*	82%
Amoebozoa	*Lamproderma ovoideum*	85%
Cercozoa	*Cercomonas plasmodialis*	99%
Apicomplexa	Eimeriidae spp.	89–99%
Dynophyceae	Heterocapsaceae	89–91%
Ciliophora	*Bresslauidea discoideus*	97%
	*Campenella umbellaria*	87%
	Colepidae spp.	95%
	*Epistylis galea*	88–93%
	*Opercularia microdiscum*	87–91%
	*Peritrichia* sp.	87–91%
	*Trithigmostoma steini*	90%

Sloths are also carriers of a variety of arthropod‐associated viruses (‘arboviruses’; e.g. phleboviruses, flaviviruses, encephalitis viruses, and orthobunyaviruses) (Seymour, Peralta & Montgomery, 1983; Gilmore *et al*., 2001; Medlin *et al*., 2016; de Oliveira Filho *et al*., 2020) and insect‐borne protozoans (e.g. trypanosomes, such as *Leishmania*) (Shaw, 1985; Gilmore *et al*., 2001; Muñoz‐García *et al*., 2019). These pathogens may be transmitted by (free‐living or epibiotic) arthropod bites to blood but may also be found on the skin or within sloth fur. Phlebotomine sandflies that reside in the fur of sloths are known carriers of *Leishmania*, which causes leishmaniasis in humans (Arias & Freitas, 1978; Herrer & Christensen, 1980; Christensen *et al*., 1982). *C. hoffmanni* sloths likely become infected by these trypanosomes in their first few months of life and remain infected for a long time, but appear asymptomatic and do not show signs of pathology (Herrer & Christensen, 1980).

## IV. FUTURE DIRECTIONS

### (1) Transmission of fur epibionts and coevolution

How are sloth fur epibionts acquired and transmitted? There are few to no data on the modes of transmission of the different sloth fur epibionts, or whether there are taxon‐specific differences in the degree of vertical *versus* horizontal transmission. To determine the extent of epibiont coevolution with sloth hosts, however, it is critical to determine the degree of vertical transmission of the epibiont community and reciprocal adaptation (Bordenstein & Theis, 2015; Meng *et al*., 2018; Rosenberg & Zilber‐Rosenberg, 2018; Roughgarden *et al*., 2018; Simon *et al*., 2019). Sloth algae, the most studied fur epibiont, is believed to be transmitted vertically from mother to baby (Beebe, 1926; Britton, 1941; Suutari *et al*., 2010) although this has not been tested directly. Horizontal transmission from environmental species pools to the sloth cannot be ruled out given the absence of data characterizing these pools. This is true for all fur epibionts that have been hypothesized to be specific to sloths. A mixed mode of epibiont transmission is likely, however, given that vertical and horizontal modes represent extreme cases (Rosenberg & Zilber‐Rosenberg, 2018). Obligate symbionts generally rely on vertical transmission (Rosenberg & Zilber‐Rosenberg, 2018), although there are also no compelling data on whether the algae on sloths are obligately or facultatively associated. It is tempting to imagine that the peculiar cracks/grooves of sloth hair may be a coevolved adaptation of the sloth with fur algae, but it is possible this may simply be a trait exapted by algae, given that cracked hair retains water that may aid sloths in thermoregulation (see Section III.1*a*).

The fact that over a third of sampled fungi from sloth fur were found to be identical to plant endophytic fungi from foliage in the same regional habitat and that other identified fungal genera can also be found in soil and with plants (Section III.3) suggests that fur microbes could in general be acquired from the environment. This may be particularly true for arthropods that are more mobile than microbes. These results highlight the importance of carefully characterizing environmental species pools to establish a null model that can be used to test hypotheses about vertical transmission or coevolution (*cf*. Fountain *et al*., 2017), efforts that have been lacking for sloth algal surveys to date. Although several arthropods appear to be specific to sloths, including the ticks *A. geayi* and *A. varium* (Waage & Best, 1985), the scarab beetle *U. gorgon* (Fig. 7), and sloth moth *C. choloepi* (Waage & Montgomery, 1976; Fig. 8), their transmission across generations remains unknown. The notion that sloth moths could have co‐evolved with *B. variegatus* and the green alga *T. welckeri* would require a high degree of partner fidelity (Archetti *et al*., 2011; Kaltenpoth *et al*., 2014). However, it is uncertain how after 3–4 weeks of maturation at a site of sloth defecation (Waage & Montgomery, 1976; Section III.2*c*) the moths emerge to find the original sloth host (rather than a different sloth) given the extent of sloth movement during this time (Section II.2).

In theory, horizontal transmission of epibionts could also occur between different sloths. However, sloths are considered solitary animals (Taube *et al*., 1999; Soares & Carneiro, 2002; S. Trull, unpublished data) and they generally do not interact with animals of other species, except for the occasional bird eating an insect off the sloth (Neam, 2015). On rare occasions, individuals from both two‐ and three‐fingered sloths have been observed to share the same or adjacent trees (Silva *et al*., 2013), including one extreme case of up to five *B. variegatus* sloths on a cacao farm feasting on the same *Cecropia* tree for over a month until the tree was completely defoliated (Vaughan *et al*., 2007), although this latter example may be far from representative of behaviour in the natural habitat. There is one brief and aggressive encounter documented in the literature between two males possibly related to defending mating territory (Greene, 1989), but this is consistent with sloths’ general preference to live alone. It is thus unlikely that sloth epibionts are typically transmitted through social contact with other sloths or other animals.

Sloths of the same species do, however, interact during two phases of sloth life history at which time fur epibionts could be transmitted: mating and early development. Sloths mate with the male on the back of the female or face‐to‐face, and can copulate for up to 7 min (Bezerra *et al*., 2008; Dias *et al*., 2009; Richard‐Hansen & Taube, 1997; S. Trull, unpublished data). Close physical contact during copulation could allow for the transmission of epibionts, especially mobile epibionts, such as arthropods, along with any microbes they might carry. Between the birth of young (gestational period of 5–10 months), sloths mate every 10–15 months for a total period of ~20 years (Taube *et al*., 2001); this amounts to approximately 10 matings over the life of a sloth, often with a different partner. The role of sex and the ‘reproductive microbiome’ – i.e. the microbiome associated with the reproductive system of parents that may inoculate offspring (Rowe *et al*., 2020) – on the transmission of fur epibionts between sloths remains to be elucidated.

Sloths give birth to their young in the canopies of trees, and newborn sloths immediately cling to the fur of the mother sloths’ abdomen for a continuous period of 5–7 months (Ramirez *et al*., 2011). Young sloths generally cling to the abdomen of their mother, not her back. However, juvenile sloths do climb onto the back and sides of the mother when she is stationary (Soares & Carneiro, 2002; S. Trull, unpublished data). It is not clear what microbes grow on the abdomen of sloths, since all sloth hair microbiome studies to date have sampled from the greenest parts of the sloth, generally the head, shoulder, or back (Suutari *et al*., 2010; Pauli *et al*., 2014). Since juvenile sloths remain on their mother for so many months, fur epibionts are likely vertically transmitted due to protracted close contact. At the very least, mothers dictate the exposure of their young to environmental species pools by the nature of their own movement throughout the forest canopy (Soares & Carneiro, 2002). Frequent sampling and analysis of sloth hair from a mother and her young throughout the period of maternal care and after the juvenile has separated from the mother would be very helpful towards resolving questions about epibiont transmission.

Sloths spend upwards of 70% of their waking hours resting in trees (Chiarello, 1998; Urbani & Bosque, 2007). They are often in direct contact with tree bark and leaves during their sleeping and resting hours, as they can be routinely found lying on branches or reclining against a branch or the trunk of a tree (S. Trull, unpublished data). Thus, transmission of biota from trees to sloths and *vice versa* is very likely, although there are few formal data to support this hypothesis. The phyllosphere is teeming with microorganisms, such as bacteria, archaea, fungi, and algae (Vacher *et al*., 2016), and by using metagenomic tools, one could in principle compare the structure and function of microbial communities on sloths and their surrounding canopy environment (Rastogi, Coaker & Leveau, 2013; Baldrian, 2017; Hassani, Durán & Hacquard, 2018). Sloths also interact with soil when they descend to the base of a tree to defecate once a week (Voirin *et al*., 2013; Pauli *et al*., 2014), where they could also acquire or disperse epibionts. The arthropods that reside in sloth fur may also be vectors that transmit microbial epibionts to and from sloths (Laroche, Raoult & Parola, 2019), especially for microbes that may have no apparent environmental species pool in the immediate surroundings of the sloth.

### (2) Sloth fur ecosystem benefits

**Fig 10:**
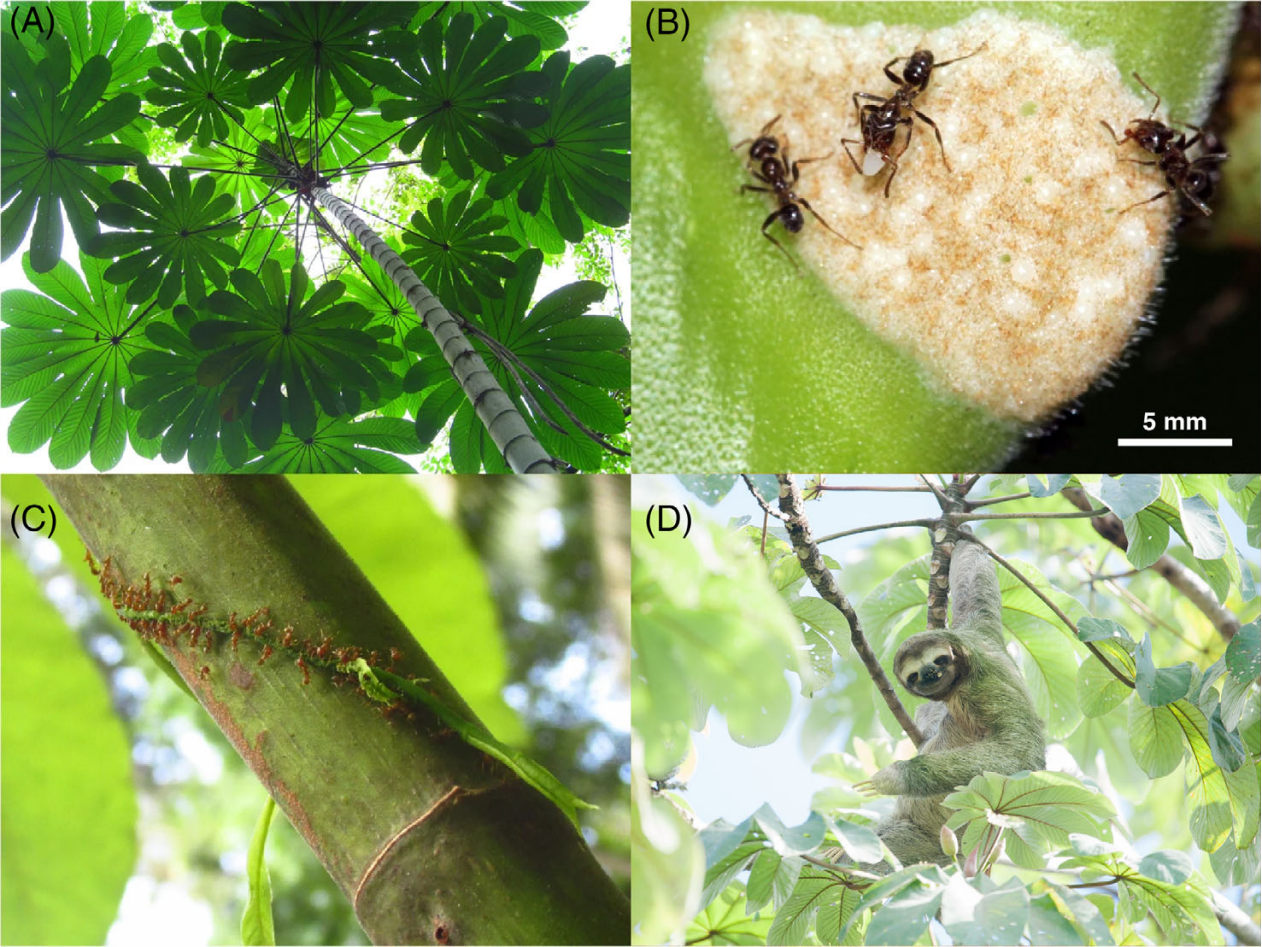
Photographs showing the (A) canopy of a *Cecropia obtusifolia* tree, (B) mutualistic ants, *Azteca constructor*, harvesting food bodies from a *Cecropia* petiole/stalk, (C) Azteca ants attacking an encroaching vine to protect a *Cecropia* tree, and (D) a brown‐throated three‐fingered sloth, *Bradypus variegatus*, eating fruit from a *Cecropia* tree, seemingly unbothered by ants. A, B, and C reproduced from Marting *et al*. (2018) with permission under Creative Commons License CC BY 4.0.

It is undeniable that sloth fur is an unusual and rich nexus of biodiversity, but does this fur ecosystem benefit the sloth in any way? Several ideas have been proposed for how algae could benefit the sloth (see Sections III.1*c* and III.2*c*); while all are problematic and/or lack empirical support, the notions that algae might provide some camouflage or chemical benefit to the sloth may be plausible. Based on these notions and inspired by the curious visual resemblance of hanging sloths to ant/termite nests (Fig. 6B), we suggest another hypothesis for how fur microbes could benefit the sloth. Azteca ants form a well‐known defensive mutualism with *Cecropia* trees (Janzen, 1969; Berg & Rosselli, 2005; Marting *et al*., 2018), a genus of trees that *B. variegatus* frequently use for food and refuge (Fig. 10; Vaughan *et al*., 2007; Ramirez *et al*., 2011; Neam & Lacher, 2015; Garcés‐Restrepo *et al*., 2019*b*). Azteca ants fiercely defend these trees from herbivores (Schupp, 1986). While it is unknown whether these ants are effective at preventing sloths from eating the leaves of the *Cecropia* (Fig. 10D), anecdotal evidence suggests that sloths are not deterred by these notoriously aggressive biting ants (S. Trull & P. Marting, unpublished data). Given the broad abilities of microbial volatile organic compounds (mVOCs) to deter or modulate insect behaviour (Davis *et al*., 2013; Engl & Kaltenpoth, 2018), it is possible that semiochemicals produced by sloth hair microbes may act to repel Azteca ants. Such mVOCs may also play a role in scent‐based sloth–sloth recognition and mating (see Silva *et al*., 2013). While mVOCs from plants (Leach *et al*., 2017), bacteria, and fungi (Dickschat, 2017; Lemfack *et al*., 2017) have been investigated, the capacity for algae to produce such compounds has been little explored (Achyuthan *et al*., 2017; Lemfack *et al*., 2017). Given the prevalence of bacteria (e.g. *Streptomyces* and *Myxobacteria*) that produce mVOCs in addition to other diverse compounds (Audrain *et al*., 2015; Lemfack *et al*., 2017; Veselova, Plyuta & Khmel, 2019) and unexplored algal and fungal mVOCs, the sloth fur microbiome may be a reservoir for novel mVOC‐producing microbes.

Microbes are known to play a fundamental role in the development and immune function of most animals (McFall‐Ngai, 2014, 2015; Bosch, Guillemin & McFall‐Ngai, 2019) and this may also be true for sloths. Sloths in captivity face many health challenges (de Stefani Munaó Diniz & Oliveira, 1999); misinformed practices at sloth rehabilitation facilities and zoos, such as bathing sloths without a specific need, could be ridding them of beneficial fur epibionts and disrupting fur ecosystem balance in a manner that negatively impacts sloth well‐being (*cf*. Hooks & O'Malley, 2017; Levy *et al*., 2017). The fungal infections afflicting wild sloths (Section III.3; Fig. 9) may serve as a practical target for understanding the basis of sloth disease regarding the normal functions and composition of the sloth fur ecosystem. Because symbiotic interactions can be context dependent and mutualists/commensals can potentially become parasites (Bronstein, 1994; Kogel, Franken & Hückelhoven, 2006; Leung & Poulin, 2008; Jones *et al*., 2015; Akçay, 2017; Vostinar & Ofria, 2019), it is possible that these fungal infections stem from resident or dormant members that become pathogenic due to environmental shifts or fur ecosystem imbalance. It is also possible that the sloth fur ecosystem endows sloths with a resilience against external pathogens.

### (3) Access to a unique ecological regime in time and space

It is becoming evident that explicit consideration of spatial, temporal, and phylogenetic scales (specifically ideas of granularity and extent) along with system nestedness will be critical to elucidating both the patterns and mechanisms of community assembly in ecosystems in which microbes play a foundational role (Addicott *et al*., 1987; Wiens, 1989; Wang & Loreau, 2014; Shade *et al*., 2018; Ladau & Eloe‐Fadrosh, 2019). For sloths that are effectively foci of epibiont biodiversity, it may be fruitful to consider how much of the sloth fur community could be understood from the perspective of island biogeography (MacArthur & Wilson, 1967; Bell *et al*., 2005; Peay *et al*., 2007; Wilson, 2010; Belisle, Peay & Fukami, 2012; Glassman *et al*., 2017; Proctor & Relman, 2017). Sloths may be a good model for examining interactions at different hierarchical levels whereby biotic feedbacks between microbes and higher trophic levels of an ecosystem are explicitly considered in understanding how host ecosystems are shaped and structured (Leibold *et al*., 2004; Carmona *et al*., 2016; Seibold *et al*., 2018; Miller, Svanbäck & Bohannan, 2018*a*; Leibold & Chase, 2019; Liu *et al*., 2019).

We have referred to the sloth as a ‘mobile ecosystem’ to highlight the fact that sloths experience life and movement within an unusual regime of time and space, unlike most other macro‐organisms. They may serve as genetic and biotic ‘mobile links’ within neotropical forests (Lundberg & Moberg, 2003). The slow movements of sloths through their geographical range and the vertical column of the forest canopy may allow us to examine an ecological and spatiotemporal regime not typically experienced by sessile (e.g. plants/trees) or significantly more mobile organisms of comparable size. Sloths may thus provide unique insights into ecological connectivity and movement ecology of wild, free‐ranging animals (see Jacoby & Freeman, 2016; Schlägel *et al*., 2020). As discussed herein, sloths can travel ≥38 m per day but typically do so in <1 h bursts of activity and commonly within a range of <2 ha (Section II.2); they can be found at various vertical heights between the forest canopy and the ground, to which they descend once a week to defecate. The curious abundance and diversity of microbes and arthropods that take up residence within the sloth pelage raises the question as to whether the uniquely slow timescales at which sloths move, their intermittent patterns of lateral movement, together with their vertical migrations, might facilitate this phenomenon. Is there some sort of temporal resonance of ecosystem processes with sloth movement dynamics that facilitates the striking biodiversity on sloth fur? How does community diversity change as a function of the characteristic timescales of underlying assembly/dispersal processes, animal movement, and forest structure?

The movement of sloths throughout their range and up and down the canopy column may connect and disperse fur epibionts between very different ecological niches. As sloths are scattered across the tree canopy, finding, catching, and studying sloths can be experimentally challenging. However, with recent advances in Global Positioning System (GPS) tracking and remote‐sensing/monitoring technology (Kays *et al*., 2015; Lennox *et al*., 2017; Neethirajan, 2017; Taylor *et al*., 2017; Hughey *et al*., 2018; Shipley *et al*., 2018; Williams *et al*., 2019; Ripperger *et al*., 2020), it may now be easier and more feasible to pursue continuous monitoring studies of sloths that are otherwise difficult to investigate by traditional search‐and‐catch methods. These capabilities may make sloths – along with their entourage of microbial and arthropod epibionts – a tractable model for exploring questions of context dependency given habitat differences across their large geographical range. Accurate time‐resolved data of sloth movements in three dimensions (latitude, longitude, and altitude/elevation) are currently lacking, limiting a deeper understanding about how sloths move through the forest, their interactions with their environment and other animals, and their responses to habitat degradation or change (Pool *et al*., 2016; Santos *et al*., 2016; Brandão *et al*., 2019; Garcés‐Restrepo, Pauli & Peery, 2019*a*). Data on social and habitat connectivity are critical for understanding the sources and modes of symbiont transmission. Coupling movement (spatial geo‐tracking) data with real‐time local environmental sensing (or time‐series data) of temperature, humidity, light, etc., and with periodic biodiversity surveys of sloth fur, would provide invaluable insights into the degree of variation and environmental conditions that a sloth experiences regarding how the fur ecosystem is structured and changes.

### (4) The nature and network of sloth–epibiont interactions

Thus far, few studies have even attempted to simply determine the nature of the interactions between sloths and their fur epibionts. Building upon this limited knowledge, future efforts should aim to identify the symbiotic traits of each interacting organism and the selective pressures acting on those traits. The ecosystem functions of sloths within their native habitat are largely unknown, although they are believed to be an important source of long‐term stable nutrients at the base of trees where they defecate (Montgomery & Sunquist, 1975). It will be important to determine through environmental sampling if algae like *T. welckeri* are generally limited to growth on sloths or if they can grow independently on other environmental substrates within the sloth habitat. If found environmentally, it would provide support for a model in which sloths acquire algae from the environment, and will provide a proper null model by which to assess sloth–algae coevolution as discussed in Section III.1. Other organisms are found in sloth fur, including bacteria, euglenozoans, amoebozoans, cercozoans, and alveolates (Table 4; Wujek & Lincoln, 1988; Suutari *et al*., 2010), many of which appear not to be present in the environment around sloths (Suutari *et al*., 2010). The functions of these organisms in the sloth hair ecosystem are unknown but have the potential to directly impact sloth health. Sloths appear to be carriers for several arthropod‐borne viruses and parasites and understanding the basis for why sloths seem not to be burdened by such pathogens may be of relevance to human health. Also unclear is the role that microbial epibionts have in facilitating host defence against pathogens in general, which has been well demonstrated in plant and pollinator systems (Liu *et al*., 2019).

As discussed above, the slow but unusual movement dynamics of sloths through the ‘volume’ of forest canopy may enable them to be hotspots of biodiversity. The stop‐and‐go pattern of movement of sloths and their migrations of over several m/day at relatively slow speeds may make them attractive for arthropod occupancy and dispersal *via* hitchhiking. The slow movements of sloths may also enable them to be colonized more easily by biota like lichenous fungi (being more similar to a tree than many fast‐moving animals; Supplementary Videos S1 and S2) that require low‐levels of movement disturbance. Interestingly, epizoic lichens, fungi, and/or cyanobacteria have been found to grow on arthropods, specifically two species of leaf mantis in the genus *Choeradodis* (Lücking, Mata‐Lorenzen & Dauphin, 2010) and various harvestmen arachnids (within small pits) (Machado & Vital, 2001; Proud *et al*., 2012; Young, Moore & Townsend Jr., 2018). Whether any of the sloth‐associated arthropods carry these taxa epibiotically is unknown, but fungal–algal associations in sloth fur could potentially link sloths to arthropods and bacteria.

As primary producers, photoautotrophic algae are at the base of the food web in many ecosystems (Polis & Hurd, 1995; Segovia *et al*., 2015; Kohlbach *et al*., 2016; Brocks *et al*., 2017), and are likely to serve as the base of the sloth fur ecosystem as well. Algae may be dependent on nitrogen from resident fur arthropods (*via* faeces) or nitrogen‐fixing bacteria, but there are no data available on this. It is also unclear how algal growth influences the composition of the rest of the microbiome and if arthropods farm and/or consume the algae. Microbial epibionts in sloth fur may provide supporting services, including producing ‘pioneer’ metabolite products that provide a foundation for community development, biofilm formation, nutrient cycling, and a thriving ecosystem (McKenney *et al*., 2018). As a poorly studied reservoir for potentially novel microbial and genetic diversity, these hair algae/microbes may produce specialized or secondary metabolites that prevent infections, or volatiles that repel ectoparasites/predators or attract arthropods in a similar manner to how plants use volatiles to attract or repel pollinators and predators (Kessler & Baldwin, 2001; Pichersky & Gershenzon, 2002). In so doing, these natural products may play a vital role in the chemical ecology of the fur ecosystem, in shaping epibiont community structure, and in modulating sloth scent in a beneficial manner. Microbes associated with insects are known to be a source of bioactive compounds and enzymes that have biotechnological potential (Berasategui *et al*., 2016) and sloth microbes may ultimately be of relevance to human health and agriculture.

## V. CONCLUSIONS

1. Sloths are slow‐moving, tree‐dwelling, leaf‐eaters in Central and South America that experience life and movement within an unusual regime of time and space, unlike any other macro‐organism. With a taxonomically diverse epibiotic community residing in their fur, sloths can be considered ‘mobile ecosystems’ or moving islands of biodiversity. They are unique models for investigating ecological and evolutionary processes linking microbes, arthropods, animal host movement, and neotropical forest resources and structure.
2. Sloth hair is unusual in having cracks or grooves that absorb water and support the growth of epibiotic algae, which is the basis for the distinct coloration of sloths in the wild. These primary producer algae are part of a rich multi‐trophic community of microbes (including bacteria, fungi, and protists) and arthropods (including moths, beetles, sandflies, triatomine bugs, lice, mites, and ticks). Sloths are also carriers of a variety of arthropod‐associated viruses (‘arboviruses’). To date, how the fur ecosystem assembles, functions, and the benefits it provides the sloth host are unclear at best.
3. It is not known whether sloths have coevolved with their fur epibionts nor how these epibionts are transmitted across generations of sloths. Incomplete studies to date suggest that sloths may host unique species of algae (*Trichophilus welckeri*), ticks (*Ambylomma geayi* and *Ambylomma varium*), and moths (*Cryptoses choloepi*) that rarely if ever are found on other hosts, consistent with the idea of vertical epibiont transmission. However, this hypothesis has yet to be tested empirically, and the absence of data on environmental species pools (which would support modes of horizontal transmission) is not data of absence. Sloth‐associated fungi have been found in soil and plants and over a third of fungal isolates obtained from sloth fur are identical to endophytes obtained from plants in the area, suggesting that horizontal transmission of at least some microbial epibionts may be likely.
4. Accurate animal tracking of sloths in four dimensions (latitude, longitude, altitude/elevation, and time) as they move through up, down, and across the tree canopy would provide extremely useful data for understanding the movement ecology of these mobile ecosystems as they interact with different components of the neotropical forest. Coupling movement data with real‐time environmental sensing of temperature, humidity, and light (with periodic biodiversity surveys of sloth fur) would provide particularly valuable insights.
5. Experimental efforts should focus on elucidating sloth fur ecosystem functions, the nature of epibiont–epibiont and sloth–epibiont interactions, and selective forces that shape this ecosystem. Understanding how fur ecosystem diversity and resilience impacts sloth health is an important practical target that could inform best practices on caring for sloths at rehabilitation facilities and zoos. Like other mammalian microbiomes, sloth fur microbiota may play an important part in immune function, and may also play a vital role in the chemical ecology of the fur ecosystem and of sloths with other individuals.

## Supporting information


**Video S1**. A male *Bradypus variegatus* (brown‐throated three‐fingered sloth) climbs up a tree after being released following a health check‐up at The Sloth Institute in Manuel Antonio, Costa Rica.

**Video S2**. A female *B. variegatus* (brown‐throated three‐fingered sloth) climbs up a tree after being released following a health check‐up (at The Sloth Institute in Manuel Antonio, Costa Rica) on her and her baby, seen clinging to her abdomen. Video by Carole Moncoquet (used with author's permission).

**Video S3**. A female *B. variegatus* (brown‐throated three‐fingered sloth) climbs up a tree after being released at night following a health check‐up at The Sloth Institute in Manuel Antonio, Costa Rica. Note the green coloration on her shoulders as well as the sloth moths swarming her back.

**Video S4**. A female *Choloepus hoffmanni* (Hoffmann's two‐fingered sloth) climbs relatively quickly up a vine (compared to a brown‐throated three‐fingered sloth) after a health check‐up at The Sloth Institute in Manuel Antonio, Costa Rica.
